# Late Ultra-High-Risk Recurrence of Gestational Trophoblastic Neoplasia Seven Years Posttreatment

**DOI:** 10.7759/cureus.75415

**Published:** 2024-12-09

**Authors:** Thang V Nguyen, Linh H Le, Giang T Nguyen, Tuan M Vo

**Affiliations:** 1 Gynecological Oncology, National Hospital of Obstetrics and Gynecology, Hanoi, VNM; 2 Gynecology, National Hospital of Obstetrics and Gynecology, Hanoi, VNM; 3 Obstetrics and Gynecology, Hanoi Medical University, Hanoi, VNM; 4 Obstetrics and Gynecology, University of Medicine and Pharmacy at Ho Chi Minh, Ho Chi Minh, VNM

**Keywords:** chemotherapy agents, gestational trophoblastic neoplasia, recurrent, vietnamese, β-hcg

## Abstract

Gestational trophoblastic neoplasia (GTN) comprises a category of malignant or potentially malignant tumors that arise from gestational trophoblasts. Almost all cases of GTN experience a recurrence within the first year following treatment, although recurrences become rare after five years. Recurrent GTN tends to have a poor prognosis, primarily due to challenges in management, a high rate of relapse, and a low five-year survival rate.

We documented a case of a patient with posttreatment ultra-high-risk recurrent GTN after seven years. The individual was hospitalized due to elevated serum beta-human chorionic gonadotropin (β-hCG) levels, liver metastasis, and enlarged lung size. After three cycles of the etoposide and cisplatin (EP) regimen, the patient showed a positive response before transitioning to the eight cycles of conventional etoposide, methotrexate, actinomycin D, cyclophosphamide, and vincristine (EMA/CO) protocol.

## Introduction

Recurrent gestational trophoblastic neoplasia (GTN) consists of a group of malignant or potentially malignant trophoblastic tumors. The relapse rate for patients with GTN is 6.5%, increasing to 33.3% for those who experience second and third recurrences [1]. Nearly all relapses occur within the first year, while late recurrences after five years are uncommon [2].

A crucial factor in relapse risk is the initial risk score assessed using the International Federation of Gynecology and Obstetrics (FIGO) scoring system. According to this system, a total score of ≥7 indicates high risk, while a score of ≥13 indicates ultra-high risk (the scoring considers elements such as age, gestational history, time since the previous pregnancy, pretreatment serum beta-human chorionic gonadotropin (β-hCG) levels, and characteristics of the GTN lesion). Higher FIGO scores correlate with an increased risk of posttreatment relapse. Additional factors that may contribute to recurrence include receiving fewer than two cycles of consolidation chemotherapy after achieving serum β-hCG levels below 5 IU/L, an interval longer than 12 months between the antecedent pregnancy and initiation of chemotherapy, and a delay of over 14 weeks between chemotherapy administration and normalization of serum β-hCG levels [3,4].

GTN with poor prognosis presents challenges in treatment, exhibits a high relapse rate, and has a low five-year survival rate. Multi-agent chemotherapy regimens such as etoposide, methotrexate, actinomycin D, cyclophosphamide, and vincristine (EMA/CO) are vital for enhancing remission rates and minimizing relapses [3,5]. In high-risk GTN patients, EMA/CO is oftentimes a standardized treatment approach. To mitigate early mortality in ultra-high-risk cases, guidelines from the European Society for Medical Oncology (ESMO) and the American National Comprehensive Cancer Network (NCCN) recommend administering low-dose Etoposide and Cisplatin (EP) chemotherapy regimen before transitioning to standardized multi-agent chemotherapies like EMA/CO [6,7].

We present a case of recurrent GTN diagnosed seven years posttreatment, characterized by ultra-high-risk indicators such as significantly elevated serum β-hCG levels, liver metastasis, and enlarged lung size. The patient underwent three consecutive cycles of a low-dose EP chemotherapy regimen before transitioning to EMA/CO. This occurrence is both uncommon and important, particularly after a seven-year interval, as it relates to deficiencies in the current body of literature.

## Case presentation

**Figure 1:**
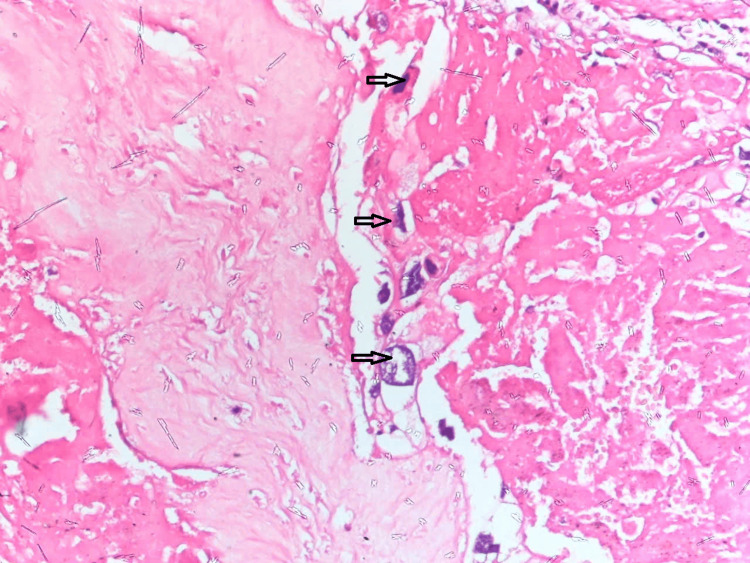
Invasive hydatidiform mole. Hysterectomy (2016): Histopathological examination revealed an invasive hydatidiform mole.

A 45-year-old female patient who had no significant personal or family medical history was diagnosed with GTN and received care at the Gynecologic Oncology Department of Central OBGYN Hospital in 2016, at the age of 38. After a total hysterectomy, a histopathological analysis identified an invasive hydatidiform mole with a FIGO score of 2 (Figure 1).

Following surgery, she began mono chemotherapy with methotrexate; however, due to treatment resistance, her regimen was switched to a multi-agent protocol known as EMA/CO. Her condition improved, as indicated by the serum β-hCG levels dropping below the threshold after four cycles; however, she received only consolidation therapy before being discharged. For the following two years, she had regular monitoring at the hospital, undergoing serum β-hCG testing. Subsequently, for the next three years, she transitioned to at-home testing every three months, conducted by a trusted medical facility, with negative serum β-hCG levels throughout the five-year follow-up. She ceased monitoring two years ago because of the COVID-19 pandemic.

**Figure 2:**
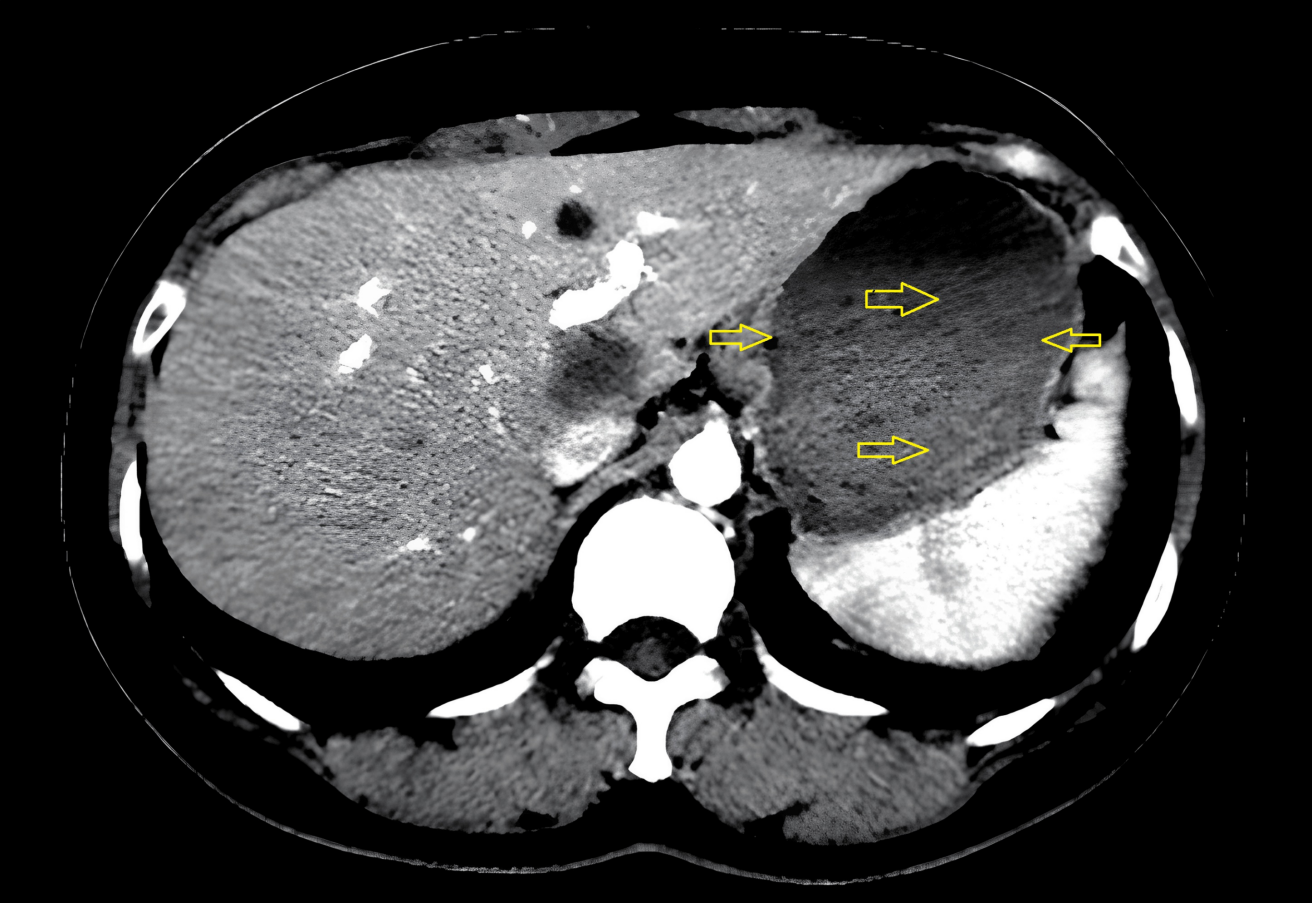
MRI of the liver revealing tumors measuring 50-54 mm in hepatic lobule VII.

**Figure 3:**
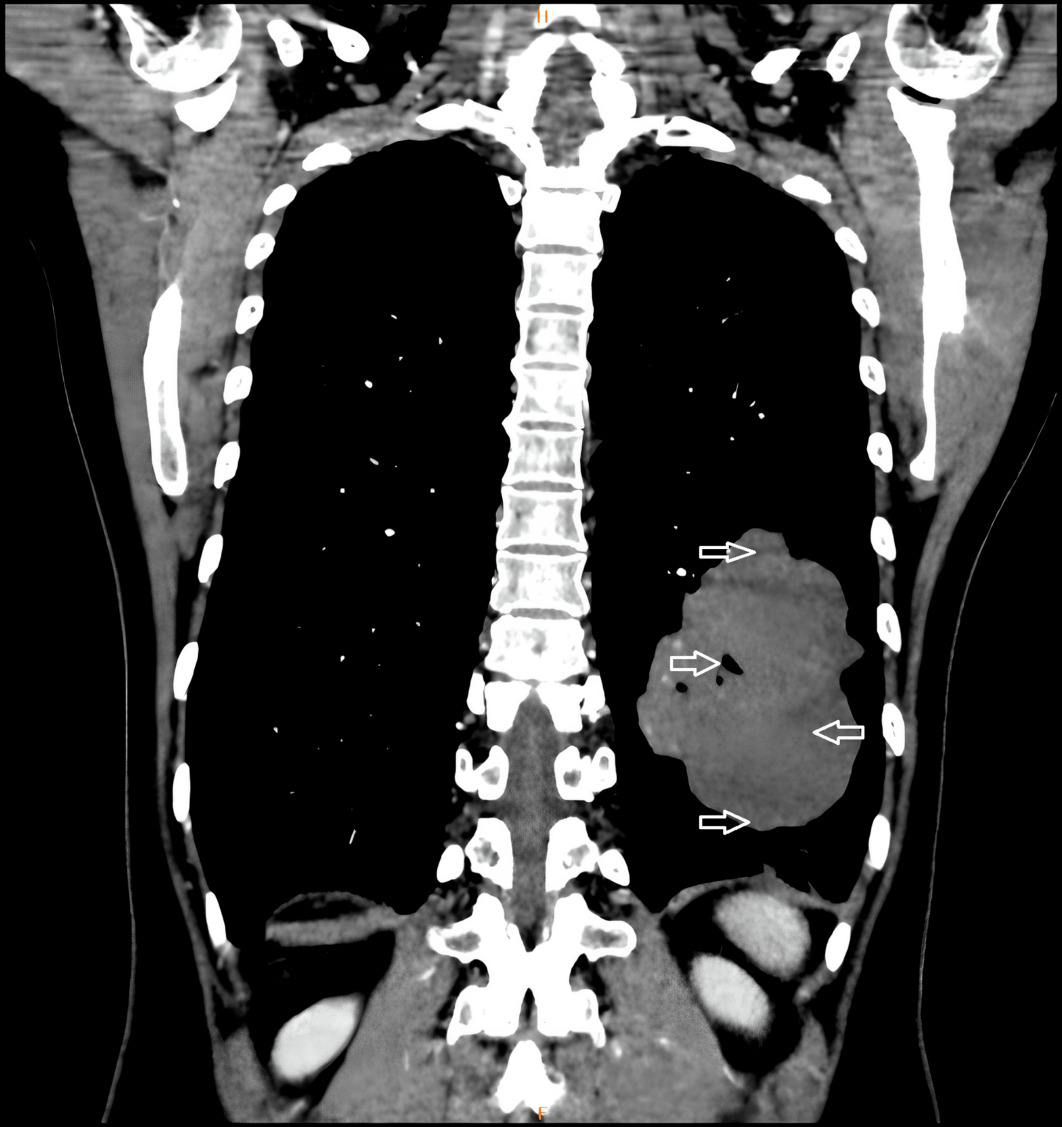
MRI of the lung showing a mass of 140 mm x 90 mm in the lower left lung.

Approximately three months before her latest hospitalization, she suffered from mild hypergastric discomfort and significant tiredness. Upon her admission, she was conscious and conveyed experiencing dull pain in the upper abdominal area, without any indication of vaginal bleeding. Her vital signs were as follows: blood pressure (BP) 110/75 mmHg, pulse rate (PR) 84 bpm, and temperature (T) 36.5 °C. Laboratory tests were normal, but the serum β-hCG level was notably high at 900,938 IU/L. Liver function tests and alpha-fetoprotein (AFP) were within normal limits, and she tested negative for hepatitis B surface antigen (HBsAg) and HIV (Table 1). Imaging revealed tumors measuring 50-54 mm in hepatic lobule VII via MRI (Figure 2) and a mass of 140 mm x 90 mm in the lower left lung on chest X-ray and MRI as well (Figure 3), with no abnormal findings in the brain MRI.

**Table 1:** Lab tests on admission. Test results on admission (5/2023), first time after seven-year relapses. β-hCG, beta-human chorionic gonadotropin; SGOT, serum glutamate oxaloacetate transaminase; SGPT, serum glutamate pyruvate transaminase

Lab tests	Results	References
Red blood cell	3.92 M/L	4.0-5.2 M/L
White blood cell	13.5 G/L	4-10 G/L
Hemoglobin	102 G/L	150-450 G/L
Platelets	460 G/L	120-160 G/L
SGOT	42 UI/l	<35 UI/L
SGPT	14 UI/L	<35 UI/L
Urea	5.2 mmol/L	2.5-6.7 mmol/L
Creatinine	60 μmol/L	50-98 μmol/L
β-hCG	900,983.00 UI/L	<5.3 UI/L

The diagnosis of ultra-high-risk recurrent GTN was established, given a FIGO score of 21, indicating a significant mortality risk due to potential metastatic node rupture. A decision was made to initiate a low-dose EP regimen for three consecutive cycles before transitioning to the eight cycles of standardized EMA/CO regimen.

Following three cycles of EP, the patient showed good responsiveness, with serum β-hCG levels decreasing from 900,938 to 15,004 IU/L. Throughout this period, her overall condition remained stable without complications such as metastatic node rupture, infection, or anemia, and blood test results (aspartate aminotransferase [AST], alanine aminotransferase [ALT], total bilirubin, direct bilirubin, and serum creatinine) remained normal after each treatment cycle.

## Discussion

The patient represents an unusual case of late recurrent GTN occurring seven years posttreatment. Research by Kong et al. involving 1,827 GTN cases treated at League University Hospital from 2004 to 2017 indicated that the average time to relapse was three months, with a significant majority (78.8%) of recurrences occurring within one year following treatment completion. Additionally, only two cases of recurrent GTN were reported after five years of monitoring [4]. Recent studies from the United Kingdom involving 4,201 patients showed that the highest risk of relapse occurred within the first year after treatment, with this risk decreasing over time; no relapses were observed beyond seven years. Consequently, some researchers argue that lifelong monitoring of β-hCG levels may be unnecessary and suggest discontinuing monitoring after 10 years [2].

There have been case reports of GTN in menopausal women. In Kong et al.'s study, over 10% of patients experienced a relapse after two years [4]. Our patient, during a five-year review, had a serum β-hCG level below the diagnostic threshold but chose to discontinue monitoring without her physician’s advice. As a result, seven years later, she was hospitalized in a high-risk GTN state with multiple metastases to various organs.

This case highlights the necessity for lifelong monitoring as recommended by ESMO and NCCN. In the United Kingdom [7,8] posttreatment monitoring involves weekly hCG tests for six weeks after chemotherapy, followed by biweekly serum and urine tests for six months, then switching to urine assessments - initially monthly, then biannually after five years.

Regarding this patient’s relapse factors, we noted that her initial risk score during chemotherapy was low (score 4), but she was resistant to methotrexate, necessitating a change to the EMA/CO regimen and the addition of a unique consolidation cycle. Her relapse was exacerbated by late detection due to discontinued monitoring, leading to various symptoms, including syncope, headache, hemoptysis, cough, and pleural pain, all related to metastatic lesions in the brain, liver, and lungs. The risk of fatality was significant due to the potential rupture of these lesions, along with the threat of treatment failure. Such complications often arise in patients who discontinue follow-up, emphasizing the critical importance of counseling on the necessity of ongoing posttreatment monitoring [3].

For high-risk GTN patients, EMA/CO is the standard chemotherapy regimen. Alternatives include paclitaxel and cisplatin or paclitaxel and etoposide (TP/TE) and bleomycin, etoposide, and cisplatin (BEP). Recently, ESMO and NCCN recommended using a low-dose regimen of etoposide (100 mg/m²) and cisplatin (20 mg/m²) for the first two days, repeated every seven days, to mitigate early mortality before starting EMA/CO for high-risk GTN patients [6-8].

In the context of high-risk GTN, as defined by a FIGO score of ≥7, particularly ultra-high-risk cases with a score of ≥13, this approach allows for gradual tumor reduction. Lower doses are intended to reduce the risk of complications, such as bleeding or tumor lysis syndrome, associated with more aggressive multi-agent therapies like EMA/CO [8].

A retrospective study by Alifrangis et al. at Charing Cross Hospital (1979-2010) found that high- and ultra-high-risk patients initially treated with a low-dose EP regimen had an early death rate of only 0.7% compared to 7.2% in pre-1995 patients [9]. Chan Wah Hak et al. reported a higher remission rate in patients receiving low-dose EP as primary therapy (71.4%) compared to those receiving EMA/CO (58.8%) [8]. Additionally, a study at the Central OBGYN Hospital from April to November 2021 successfully treated five ultra-high-risk GTN patients (FIGO score ≥13) with three consecutive cycles of low-dose EP before switching to EMA/CO. None of these patients experienced complications such as metastatic node rupture, infection, or anemia, and serum β-hCG levels decreased by over 97% after three cycles [10].

The patient in this case report was assessed as ultra-high risk, with a FIGO score of 21, and faced the risk of metastatic nodal rupture. Thus, low-dose EP was recommended for three consecutive cycles before transitioning to the eight cycles of standard EMA/CO regimen. She responded well to the treatment, with serum β-hCG levels rapidly decreasing after three cycles of low-dose EP, and no severe complications were observed.

## Conclusions

Recurrent GTN frequently manifests shortly after treatment, mainly within the initial year. Nonetheless, certain individuals may experience delayed recurrences, with our case extending up to seven years, highlighting the necessity for ongoing monitoring posttreatment for early identification. Implementing a sensitive low-dose EP regimen for patients categorized as high-risk and ultra-high-risk GTN before commencing standard multi-agent chemotherapy, such as EMA/CO, can help decrease the likelihood of early mortality, especially in patients with multiple metastatic lesions. 
